# Computational Modeling and Evaluation of Potential mRNA and Peptide-Based Vaccine against Marburg Virus (MARV) to Provide Immune Protection against Hemorrhagic Fever

**DOI:** 10.1155/2023/5560605

**Published:** 2023-04-17

**Authors:** Faisal F. Albaqami, Ali Altharawi, Hassan N. Althurwi, Khalid M. Alharthy, Muhammad Qasim, Ziyad Tariq Muhseen, Muhammad Tahir ul Qamar

**Affiliations:** ^1^Department of Pharmacology, College of Pharmacy, Prince Sattam Bin Abdulaziz University, Al-Kharj 11942, Saudi Arabia; ^2^Department of Pharmaceutical Chemistry, College of Pharmacy, Prince Sattam Bin Abdulaziz University, Al-Kharj 11942, Saudi Arabia; ^3^Department of Bioinformatics and Biotechnology, Government College University Faisalabad (GCUF), Faisalabad 38000, Pakistan; ^4^Department of Pharmacy, Al-Mustaqbal University College, Hillah, Babylon 51001, Iraq

## Abstract

A hemorrhagic fever caused by the Marburg virus (MARV) belongs to the *Filoviridae* family and has been classified as a risk group 4 pathogen. To this day, there are no approved effective vaccinations or medications available to prevent or treat MARV infections. Reverse vaccinology-based approach was formulated to prioritize B and T cell epitopes utilizing a numerous immunoinformatics tools. Potential epitopes were systematically screened based on various parameters needed for an ideal vaccine such as allergenicity, solubility, and toxicity. The most suitable epitopes capable of inducing immune response were shortlisted. Epitopes with population coverage of 100% and fulfilling set parameters were selected for docking with human leukocyte antigen molecules, and binding affinity of each peptide was analyzed. Finally, 4 CTL and HTL each while 6 B cell 16-mers were used for designing multiepitope subunit (MSV) and mRNA vaccine joined via suitable linkers. Immune simulations were used to validate the constructed vaccine's capacity to induce a robust immune response whereas molecular dynamics simulations were used to confirm epitope-HLA complex stability. Based on these parameter's studies, both the vaccines constructed in this study offer a promising choice against MARV but require further experimental verification. This study provides a rationale point to begin with the development of an efficient vaccine against Marburg virus; however, the findings need further experimental validation to confirm the computational finding of this study.

## 1. Introduction

Marburg virus (MARV) causes hemorrhagic fever in humans and belongs to the *Filoviridae* family and the genus Marburg virus [1]. It is classified as a risk group 4 pathogen by the World Health Organization (WHO) and can be used as a bioterrorism agent according to Center for Disease Control (CDC) [2, 3]. MARV, like all mononegaviruses, comprises of noninfectious, nonsegmented, and negative-sense ssRNA genomes with reverse complementary 3′ and 5′ termini, no 5′ cap, no polyadenylation, and no covalently attached protein [4]. MARV genomes are about 19 kbp large that are comprised of 7 genes; the genome is arranged into 3′-UTR followed by proteins in the given sequence NP, VP35, VP40, GP, VP30, VP24, and L, and at the end, there is a 5′-UTR [5]. Seven structural proteins make up Marburg virions. The helical ribonucleocapsid protein at the center consists of the MARV RNA intertwined around the nucleoprotein (NP) polymer. The RdRp or L protein with polymerase cofactor (VP35) and a transcription activator is associated with the ribonucleoprotein (VP30). The minor (VP24) and major (VP40) matrix proteins form a matrix around the ribonucleoprotein. These particles are encased in a lipid membrane created from the membrane of the host cell. A glycoprotein (GP1, 2) attaches to the surface and sends off spikes that are 7 to 10 nm in length [6]. MARV is antigenically distinct from Ebola virions, while having a structure that is substantially identical to Ebola virions.

The virus can spread between people via body fluids via unprotected intercourse and open wounds. It can cause similar symptoms as in Ebola virus fever and can cause bleeding (hemorrhage) and fever symptoms. In 1967, the MARV was first described [7]. The virus was identified after a series of epidemics in the Frankfurt and Marburg cities of Germany, as well as the Belgrade, the capital city of Yugoslav, that year [8]. Regular outbreaks of the virus have been reported worldwide since its identification, including in major parts of the world, including Europe and Africa. Most recently, MARV was reported in Guinea, where the virus was isolated on the 2^nd^ of August 2021 from multiple patients who died [9].

There is no way to treat the virus after infection, and early treatment of symptoms like dehydration greatly increases the odds of survival [10]. Ebola and Marburg vaccine extended clinical trials started in 2009 in Kampala, Uganda [11, 12]. But to date, no effective treatment or approved vaccine is in the market against the MARV. Reverse vaccinology approach has surfaced as a hopeful solution to the shortcomings of classical vaccines. Conventional vaccine designs still require development in order to better comprehend their impact on human immune systems. Numerous immune-related issues regarding newly developing human viral diseases should be taken into account [13]. Targeted adaptive immune reaction activation is enabled by advanced reverse vaccinology methods. Numerous vaccines against human infections have been designed using the epitope prediction approach. This involves creating a possible vaccine candidate against “Plasmodium vivax” based on AMA-1 epitope. Recently, in 2019, a computationally designed vaccine comprising of multiple potent epitopes joined together was tested in mice, and the vaccine turned out to be inducing high IgG antibodies against *Acinetobacter baumannii*. Likewise, to this, the effectiveness of theoretically predicted B cell epitopes in detection against *Trypanosoma vivax* was also validated in wet lab experimentation. For instance, similar strategies have been used against a number of human fatal viruses, such as the Crimean-Congo hemorrhagic fever virus, Ebola, and Mokola rabies virus [14–16]. The time and expense involved in developing vaccines have significantly lowered. The efficacy and safety of the vaccines created using computational methods have been established. Human immunodeficiency virus 1, human norovirus, herpes simplex 1 and 2, *Staphylococcus aureus*, *Shigella* spp., and Ebola virus vaccines have all been developed using immunoinformatics method [17, 18]. In nonhuman primates (NHP), the Ad26.Filo and MVA-BN-Filo have shown promising immune activity in regions that are at a high risk of an outbreak of filovirus [19].

In this scientific investigation, predictions of linear B cell and T cell epitopes obtained from MARV proteins were carried out and investigated as viable possibilities for constructing two vaccine candidates, i.e., multiepitope subunit vaccine (MSV) and mRNA vaccine (MRV). Using in silico molecular docking methods, specific T cell epitopes were evaluated for physiochemical properties and interaction with their respective HLAs. The physical and chemical characteristics of MSV were also computed; then, secondary and tertiary structure modeling was done. The revised 3D model's affinity for binding toll-like receptors was assessed using a variety of immunoinformatics methods. This study may open the door to the creation of dynamic and effective vaccines that include a special association of several MARV protein-derived antigenic peptides that play various functions throughout the lytic stage of MARV infection.

## 2. Methodology

### 2.1. Collection of Proteins

**Figure 1:**
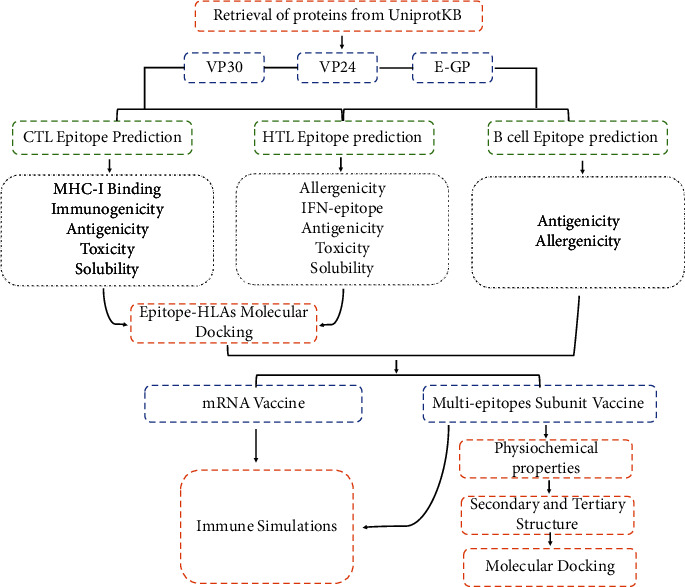
Methodological workflow of designing mRNA and multiepitope subunit vaccine against MARV in this study.

Three proteins (VP24, envelope glycoprotein, and VP30) of MARV were downloaded in FASTA format from the Universal Protein Knowledgebase (https://www.uniprot.org/) [20]. The proteins selected for vaccine designing were examined for antigenic potential utilizing an online server, VaxiJen [21]. According to a study, VP24 showed membrane-binding abilities and was enlisted into filamentous virus-like particles (VLPs) that are brought on by VP40. Additionally, when VP24 was suppressed in cells infected by MARV, using small interfering RNA (siRNA) technology, viral transcription and replication were unaffected, but the release of viral particles was drastically decreased. This provides more evidence that VP24 is necessary for a step that occurs after transcription and replication but before the budding of viral offspring. The development of transport-capable nucleocapsid and/or their interaction with the plasma membrane's budding sites are both thought to need VP24. Furthermore, VP30 is a transcriptional activator and is required for pathogenesis thus making it a validated vaccine target [22, 23]. The methodological pipeline followed in this scientific study is provided in Figure 1.

### 2.2. Prediction of T Cell Epitopes

Online servers were utilized for identification of T cell epitopes. CTL epitopes were identified by NetCTL 1.2 server at a default threshold of 0.75. The CTL predictions using NetCTL 1.2 demonstrate essential information regarding different parameters by using a weight matrix. NetCTL 1.2 has an over 0.72 sensitivity among the five percent of top-scoring short peptides in a large standardized computations including 216 HIV epitopes covering all 12 confirmed HLA super types.

### 2.3. Helper T Cell Epitope Prediction

HTL epitopes for the MARV proteins were predicted by IEDB MHC-II considering the reference set of seven HLAs using default parameters to enable the prediction of epitopes across the largest feasible population. Since HLA alleles vary greatly in their susceptibility and sensitivity among populations, it is impossible to establish a causal relationship between the prevalence of MARV and any particular HLA allele. Each HTL epitope is given an IC50 value by the IEDB MHC-II module, which is inversely related to binding affinity of the peptide to MHC-II receptor. High binding affinity is indicated by an IC50 score of smaller than 50 nM, moderate binding affinity is shown by a score of less than 500 nM, and low binding affinity is indicated by a score of less than 5000 nM for an epitope to the MHC-II receptor. The less percentile ranks the stronger binding affinity for MHC. Numerous important parameters were considered to identify HTL epitopes fulfilling criterion that will be further used for mRNA vaccine designing. Furthermore, to select the most suitable CTL and HTL epitopes of the predicted pool, multiple parameters were analyzed for individual epitopes derived from the selected proteins [14, 24–26].

### 2.4. T Cell Epitope Prioritization

High-throughput epitope screening and computational epitope prediction are two key approaches in immunoinformatics for identifying potential epitopes for vaccine development. In general, a combination of both HTL and CTL epitopes can be included in a vaccine to provide both humoral and cell-mediated immune protection. HTL epitopes are recognized by helper T cells and are crucial for generating antibody responses, while CTL epitopes are recognized by cytotoxic T cells and are critical for destroying infected cells. However, the selection of epitopes ultimately depends on the specific pathogen and the immune response that is needed to provide protection against it. Multiple parameters required for an ideal vaccine were considered to select the most suitable T cell epitopes.

#### 2.4.1. Antigenicity

VaxiJen server predicted the antigenic capacity of both CTL and HTL epitopes [21]. Instead of employing alignment techniques, the server computes the antigenic potential of a specific peptide based on its physiochemical characteristics with an accuracy of 70 to 89%. Epitopes demonstrated antigenic scores > 0.4 and were processed for further analysis.

#### 2.4.2. Immunogenicity

The epitope should have the capacity to elicit a strong and specific immune reaction in the host. Immunogenicity is the capacity of an epitope to provoke an adaptive immune reaction. The IEDB MHC-I immunogenicity module was utilized to predict the immunogenic potential of MARV-derived CTL epitopes. Epitopes scoring higher than zero were classified as immunogenic and studied further [27].

#### 2.4.3. Solubility and Toxicity

The antigenic and immunogenic epitopes were assessed for solubility and toxicity using CamSol Intrinsic and ToxinPred servers, respectively [28]. The epitope should not cause any harmful effect to the host, meaning it should not be toxic. ToxinPred evaluates multiple physiochemical characteristics of a peptide to calculate toxicity [29]. The soluble and nontoxic epitopes were selected for downstream screening.

#### 2.4.4. MHC-I Binding

The epitopes included in the vaccine should bind with high affinity to the MHC molecules, which present the epitopes to T cells. MHC class I alleles from the shortlisted CTL epitopes were identified using the IEDB server's consensus algorithm and IEDB MHC-I binding tool [30].

#### 2.4.5. Allergenicity and IFN Epitopes

HTL epitopes satisfying the above-described constraints were subjected to allergenicity prediction using Algpred2.0 server [31]. Algpred2.0 is an upgraded version of the first Algpred server created in 2006; several additional features have been included to enhance the functionality of the approach. Only the nonallergic HTL epitopes were referred for further investigation. IFN epitope servers were used to ensure that the shortlisted HTL epitopes would not induce an allergic response and be capable of inducing an interferon-gamma response, respectively [32]. The IFN epitope server is based on motif and SVM hybrid algorithm techniques for the calculation of an epitope interferon-producing capability. HTL epitopes that can prompt IFN-gamma reaction and satisfy the before-mentioned features were chosen for mRNA vaccine construction.

#### 2.4.6. Population Coverage

Population coverage is a significant consideration in in silico vaccine design, since it helps to ensure that the vaccine provides broad protection against a pathogen, and is effective in a diverse range of individuals. The chosen T cell epitopes in the mRNA vaccine construct should efficiently cover highly populated countries across the globe. The chosen T cell epitopes were examined (country as well as region-wise) using default parameters set in IEDB population coverage tool to assure that the proposed vaccination will cover the majority of the global population [33]. The above-mentioned population coverage estimation tool estimates the average coverage of epitopes in distinct peoples based on binding affinity of individual epitopes to respective HLAs. Since MV infection is a global problem, a global population coverage option was selected.

### 2.5. Linear B Cell Epitope Prediction

Linear B cell epitopes are a useful component of vaccine design, as they are easy to identify and design and highly immunogenic and can help to generate a diverse range of antibodies that target different regions of the pathogen. The kernel approach is a novel technique used by the BCPred service to estimate linear B cell epitopes [34]. The AllertopV2.0 and VaxiJen servers (cut − off = 0.4) were used to assess the antigenicity and allergenicity of the linear B cell epitopes. To design a vaccine formulation, the antigenic and nonallergic LBL epitopes were chosen.

### 2.6. Molecular Docking of Epitopes and HLAs

Molecular docking of epitopes with HLAs is a crucial step in computational vaccine design, as it enables the estimation of how epitopes will bind to HLA molecules and can help to identify the most promising candidates for inclusion in a vaccine. Selected T cell epitopes were docked in silico with corresponding human leukocyte antigens using the CABS-dock platform. The server does not need a predetermined peptide/epitope 3D structure; thus, all that was required was a PDB file of the receptor and the epitope sequences. The RMSD (root-mean-square deviation) score of the epitope/peptides was computed by CABS-dock. The ligand-RMSD score indicates the quality of the docking pose. The RMSD score is directly related to the quality of ligand-receptor binding, and a smaller RMSD score indicates better key binding contacts, indicating a good docking position. High-quality predictions have RMSD scores of less than 3, whereas moderate-quality predictions have RMSD scores between 3 and 5.5. The respective HLAs were retrieved from RCSB. Furthermore, HawkDock server was employed to estimate the binding free energy for the best conformation determined by CABS-dock server [35, 36].

### 2.7. Vaccine Sequence Construction

In this study, two vaccine candidates were devised to make use of the shortlisted T and B cell epitopes.

#### 2.7.1. Multiepitope mRNA Vaccine

A computationally designed mRNA vaccine consists of several components that are designed and optimized using bioinformatics and computational approaches. By optimizing the antigen sequence, noncoding regions, delivery system, adjuvants, and other components, it is possible to create a highly effective and specific vaccine against a target pathogen or disease. The presence of five components is considered essential of an ideal mRNA vaccine. (1) It should have 5 prime and 3 prime UTR regions, (2) a Kozak sequence should be there, (3) the vaccine structure should also have efficient B and T cell epitopes, (4) also, suitable linkers should be used to combine the epitopes, and (5) a stop codon at the end that tells the translation machinery where to stop. The AUG codon has to be included within the Kozak sequence [37], although stop codon can be enhanced [38]. Linkers are used to join epitopes in a multiepitope vaccine in order to optimize the spacing and orientation of the epitopes for improved immunogenicity. The properties of linkers used to join the epitopes in a multiepitope vaccine should be carefully chosen to ensure that the vaccine is stable, immunogenic, and nontoxic. The linkers should be flexible, rigid, and cleavable because it allows different components of the vaccine to avoid contact and prevent formation of neoepitopes [39]. According to previous studies [40, 41], the aforementioned criteria were satisfied while selecting linkers. The vaccine sequence was constructed by placing AAY, PMGLP, and GGGGS linkers to connect CTL, HTL, and linear B cell epitopes, respectively [42–44].

The tissue plasminogen activator (tPA) (UniProt ID: P00750) secretory signal sequence that has been reported to be helpful in increasing antigen presentation and release of translated epitopes was included in the vaccine sequence [45]. Since mRNA vaccines are vulnerable to instability, including elements present in mRNAs of eukaryotes is critical [46, 47]. To make the mRNA sequences stable, 5′ cap, 5′ and 3′ UTRs, and poly (A) tail were added to the vaccine. A suitable length of poly (A) tail is crucial for a stable mRNA, and too long or even too short poly (A) tails lead to inefficient translation [48]. An ideal mRNA vaccine should have a poly (A) tail around 115–150 nucleotides long, as recommended by numerous studies [49]. Poly (A) tails are known to work in tandem with 5′ m7G cap [50]. NCA-7d at the 5′ UTR and S27a+R3U at the 3′ UTR was added to the vaccine as they stabilize mRNAs [51, 52].

#### 2.7.2. Multiepitope Subunit Vaccine

Subunit vaccines are made up of antigenic sections of a pathogen that are used to initiate immune response in host. To construct the final vaccine, the anticipated T cell and linear B cell epitopes were joined sequentially. To increase immunological response, a vaccine sequence was created adding mammalian beta-defensin (a 45-mer peptide) as an adjuvant. Interactions of adjuvants with toll-like receptors (TLRs) polarize CTL responses and cause a strong immune response. TLR1, TLR2, and TLR4 receptors can all be triggered by beta-defensin adjuvant. PADRE sequence was added along with the adjuvant to help overwhelm the issues created by extremely polymorphous HLA alleles. The adjuvant and PADRE sequences were linked using EAAAK linkers. The CTL, HTL, and LBL epitopes were conjugated using AAY, PMGLP, and GGGGS linkers, correspondingly.

### 2.8. Physiochemical Property Prediction

Predicting different physicochemical properties of a vaccine is an important step in vaccine design, as it can help to optimize the stability, solubility, bioavailability, and immunogenicity of the vaccine. By optimizing these properties, it is possible to improve the effectiveness of the vaccine and increase the likelihood of a successful immune response. VaxiJen, Algpred2.0, and ProtParam servers were utilized for the calculation of the physiochemical properties (antigenicity, allergenicity, MW, PI, half-life, AI, and GRAVY) of the multiepitope's subunit vaccine.

### 2.9. Secondary and Tertiary Structure

The SOPMA predicted the secondary structure of the MSV, whereas for tertiary structure prediction, Robetta server was employed. Predicting the 3D structure of a vaccine is an important step in computational vaccine design, as it is directly connected to the stability, epitope accessibility, immunogenicity, and design of multiepitope vaccines. By optimizing the 3D structure of a vaccine, it is possible to improve its effectiveness and increase the likelihood of a successful immune response. The protein structure modeling technique used by the Robetta server includes the identification of a template model. If in case a template protein with suitable identity and similarity is identified by utilizing BLAST, PSI-BLAST, 3D-Jury, or FFAS03, the server uses the comparative modeling approach for modeling. The predicted structure was further refined using the GalaxyRefine webserver.

The validation of 3D models of vaccines is an important step in the computational design of vaccines. By ensuring the accuracy and reliability of the predicted structure, researchers can optimize the design of the vaccine for improved immunogenicity, efficacy, and safety and can support the regulatory approval of the vaccine. In this study, ERRAT, ProSA-web, and Ramachandran plot by Procheck were used to validate the quality of vaccine's 3D model generated and refined by Robetta and GalaxyRefine webservers, respectively. ERRAT assesses the non-bonded interactions within a given 3D structure. ProSA-web is commonly used to authenticate a protein 3D model and assign a quality score (*Z*-score). For instance, if the *Z*-score falls within the range determined for experimentally established protein models, the submitted protein model is most expected to have no errors. The region of the input structure, if there are problems, can be visualized on the result page of the ProSA-web server.

### 2.10. Vaccine TLR Docking and Simulations

To check the binding affinity of MSV towards Toll-like receptor, the CABS-flex algorithm was utilized for molecular docking [31]. CABS-flex, a well-suited tool for rigid-body docking protein-protein interaction that utilizes FTDock for sampling, was utilized. It gives top 10 models of protein-protein complex ranked on the basis of best score. For MSV-TLR complex with lowest energy score, residue level interaction was noted and selected top-rank models for each MSV-TLR complex. The interactions were explored using PDBsum that provides a graphical representation of the interaction pattern between both proteins. MD simulations of epitope HLA complexes can present critical understandings of the complex behavior and stability of the complex over time and can help to optimize the design of epitope-based vaccines for improved binding affinity, stability, and safety. The top two complexes from each category, i.e., HTL and CTL epitopes, were simulated using AMBER20 simulation tool. For each complex, 20 ns simulation was performed. The simulation protocol was applied as described in the previous studies [53–56].

### 2.11. Immune Simulation

Using the C-ImmSim server, computational immune simulation was performed to forecast the immune system's actual reaction to the multiepitope mRNA vaccination [57]. For the evaluation of immune system response and how epitope interact with it, this simulation model uses machine learning (ML) and PSSM, respectively. For the majority of vaccinations now in use, 4 weeks is the shortest prescribed interval between the first and second dosages [58]. For our immunological simulation, three shots were administered, spaced four weeks apart, each containing 1000 units of the vaccine. The C-ImmSim server uses a time-step scale to determine simulation run times. This scale's time steps relate to 8 hours in the actual world. The injection points were set at time steps 1, 84, and 168, correspondingly, for a total of 1050 time steps throughout the simulation. The other variables were kept with their default settings.

## 3. Results

### 3.1. Protein Retrieval for Vaccine Designing

Amino acid sequences of the envelope glycoprotein, membrane-associated protein VP24, and transcriptional activator VP30 of MARV were downloaded from the UniProtKB. VaxiJen was used to predict the antigenic potential of the proteins setting default threshold at 0.4. Antigenicity is an important parameter to estimate the ability of a protein that can induce immune response. The UniProt IDs and antigenic scores are provided in Table 1.

### 3.2. Prediction and Evaluation of CTL Epitopes

NetCTL 1.2 was utilized for prediction of CTL epitopes. The high combined score (COMB) of an epitope is directly related to binding potential to MHC-I receptors. The selected most suitable epitope multiple parameters were considered, high COMB score, immunogenicity score higher than 0, antigenicity above threshold of 0.4, and solubility above -1; and for an epitope to be classified as nontoxic, it should have a score less than 0. Four CTL epitopes that fulfilled the criterion mentioned above were selected for construction of vaccine sequence. Furthermore, IEDB MHC-I binding module predicted corresponding HLA molecules having considerable binding affinity for the selected CTL epitopes. The selected CTL epitopes with HLAs and scores are provided in Table 2.

### 3.3. Prediction and Evaluation of HTL Epitopes

HTL epitopes are bind to MHC-II receptors and activate helper T cells of host's immune system. IEDB MHC-II predicted HTL epitopes that corresponds to a reference set of 7 human leukocyte antigens or shortly as HLAs. Epitopes that have lowest adjusted percentile rank indicate higher binding affinity of that epitope to the MHC-II receptor. Multiple parameters with respective thresholds were considered to select HTL epitopes suitable for vaccine construction. Epitopes with low adjusted percentile score, antigenicity above 0.4, immunogenicity greater than 0, solubility score greater than -1, allergenicity score lower than 0.3, epitope having the ability to induce interferon-gamma response, and toxicity score lower than 0 were selected for vaccine construction. Overall, five HTL epitopes were selected based on the aforementioned criterion. The selected HTL epitopes with corresponding scores are provided in Table 3.

### 3.4. Population Coverage

**Figure 2:**
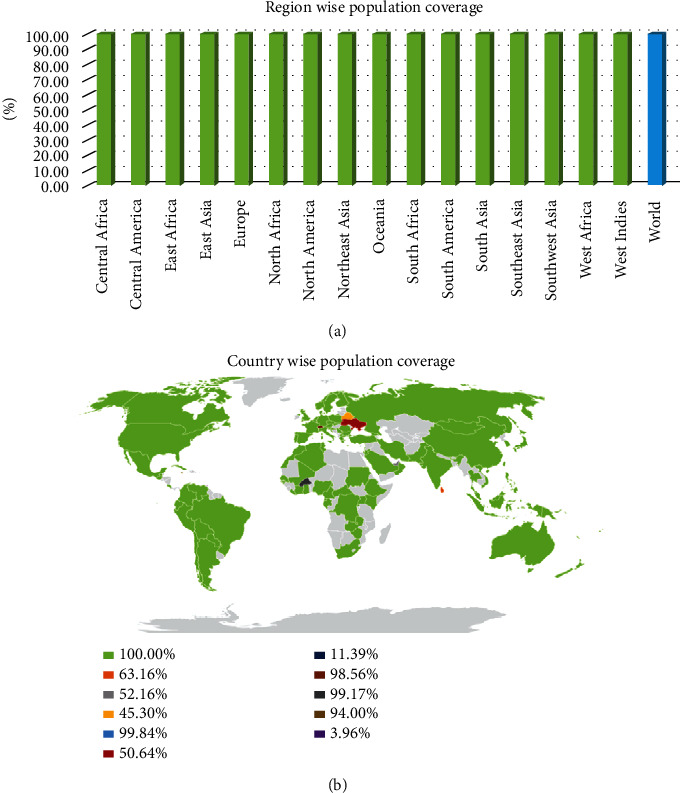
(a) Region-wise population coverage of the selected T cell epitopes shows 100% population coverage worldwide and in selected regions of the world. (b) Country-wise population coverage of the selected T cell epitopes shows 100% population coverage for the 110 countries among all the countries where data was available.

The population coverage analysis was carried out using IEDB population coverage tools. The complete set of HLAs available in the IEDB database was used to evaluate and select T cell epitopes covering most of the population worldwide. The overall results suggest the selected T cell epitopes covering 100% of the population worldwide; furthermore, region-wise population coverage had similar results (Figure 2(a)). Moreover, country-wise population coverage estimation revealed the selected T cell epitopes covering all the densely populated countries of the world where the population coverage ranged from 3.96% (Trinidad and Tobago) to 100% (110 countries). The country-wise population coverage data is provided in Figure 2(b).

### 3.5. Prediction and Evaluation of Linear B Cell Epitopes

Linear B cell epitopes are crucial for an ideal vaccine; in our study, we predicted linear B cell epitopes using BCPreds. Linear B cell epitopes with high scores are directly related to strong binding to respective receptor present on B cell surface. Screening of the epitopes was based on multiple scores, high binding score, antigenicity above threshold of 0.4, and allergenicity above the threshold level of <0.3. Six epitopes were selected to be included in the final vaccine construct. The selected epitopes with respective scores are presented in Table 4.

### 3.6. Peptide-HLA Molecular Docking

**Figure 3:**
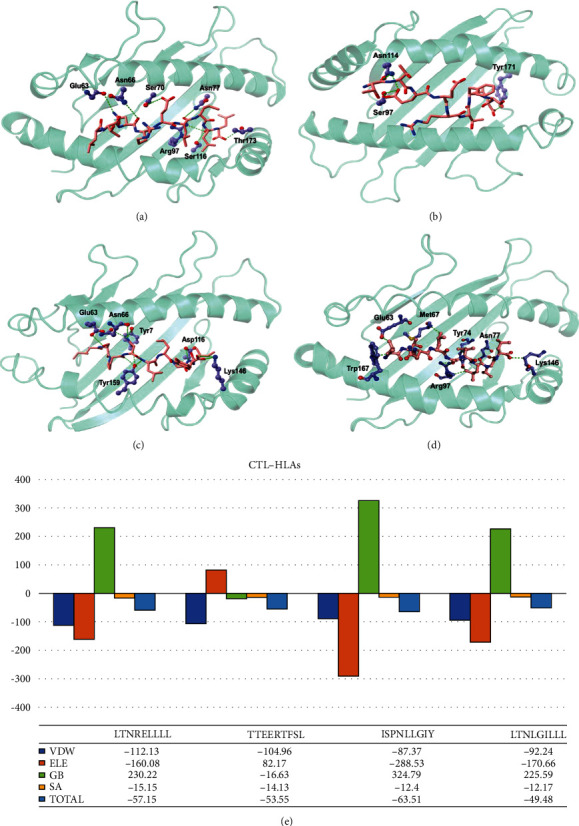
Selected CTL epitope (pink) docking complex with respective HLAs (cyan). (a) HLA-B^∗^58:01-LTNRELLLL, (b) HLA-B^∗^08:01-TTEERTFSL, (c) HLA-A^∗^01:01-ISPNLLGIY, and (d) HLA-B^∗^58:01-LTNLGILLL. (e) The binding free energy of selected CTL epitopes in complex with respective HLA molecules.

**Figure 4:**
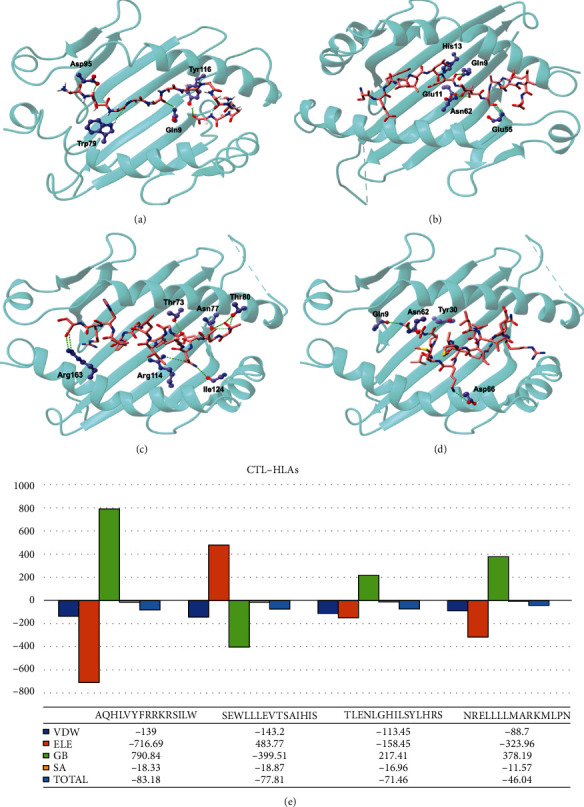
Selected HTL epitopes (pink) docking complex with respective HLAs (cyan). (a) HLA-DRB1^∗^15:01-AQHLVYFRRKRSILW, (b) HLA-DRB1^∗^07:01-SEWLLLEVTSAIHIS, (c) HLA-DRB1^∗^15:01-TLENLGHILSYLHRS, and (d) HLA-DRB1^∗^15:01-NRELLLLMARKMLPN. (e) The binding free energy of selected HTL epitopes in complex with respective HLA molecules.

The list of HLAs with corresponding PDB IDs is presented in Table 5. Eight HLAs were downloaded from RCSB, i.e., HLA-B^∗^08:01 with PDB ID: 7NUI, HLA-B^∗^58:01 with PDB ID: 5VWH, HLA-A^∗^01:01 with PDB ID: 4NQX, HLA-DRB1^∗^07:01 with PDB ID: 6BIJ, and HLA-DRB1^∗^15:01 with PDB ID: 1BX2, and prepared for docking. The total binding free energy for HLA-B^∗^08:01-TTEERTFSL was reported to be -53.55 kcal/mol, -49.48 kcal/mol for HLA-B^∗^58:01-LTNLGILLL, -57.15 kcal/mol for HLA-B^∗^58:01-LTNRELLLL, and -63.51 kcal/mol for HLA-A^∗^01:01-ISPNLLGIY. The docked complexes (CTL) are shown in Figures 3(a)–3(d) whereas the BFE is given in Figure 3(e). The BFE for the HLA complexes are HLA-DRB1^∗^15:01-AQHLVYFRRKRSILW_425-439_ (-83.18 kcal/mol), -77.18 kcal/mol for HLA-DRB1^∗^07:01-SEWLLLEVTSAIHIS_123-137_, -46.04 kcal/mol for HLA-DRB1^∗^15:01-NRELLLLMARKMLPN_103-117_, and-71.46 kcal/mol for HLA-DRB1^∗^15:01-TLENLGHILSYLHRS_150-164_. The docking pattern and BFE are given in Figures 4(a)–4(e).

**Figure 5:**
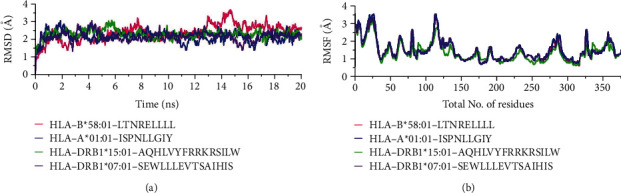
Dynamic stability calculated as RMSD (a) while residual flexibility calculated as RMSF is given in (b).

Molecular simulation of complex revealed stable dynamics calculated as root-mean-square deviation (RMSD). Each complex shown in Figure 5(a) demonstrated a deviation within 2 Å with no significant structural perturbation that concludes the stable binding of these epitopes. On the other hand, given in Figure 5(b), the residual flexibility is calculated as similar fluctuation pattern.

### 3.7. Vaccine Construction

In this study, two vaccine candidates were constructed using the selected set of 4 CTL, 4 HTL, and 6 LBL peptides:

1. An adjuvant multiepitope subunit vaccine must be cloned, expressed, purified in vitro, and injected to the host subcutaneously
2. A self-adjuvant mRNA vaccine must be produced in vitro, delivered to the host via an appropriate delivery system, i.e., nonviral lipid nanoparticles, administrated intramuscularly, and expressed in vivo

#### 3.7.1. mRNA Vaccine Construct

**Figure 6:**
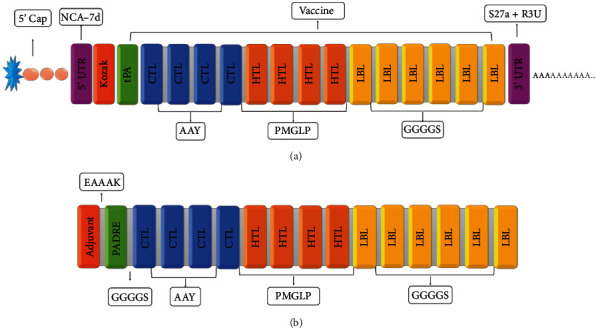
(a) The final mRNA vaccine construct. (b) The final MSV construct with different linkers.

A cluster of epitopes joined together through AAY, PMGLP, and GGGGS linkers was used to construct the vaccine candidate. The final included CTL, HTL, and B cell epitopes are presented in Tables 2–4, respectively. The 5′ m7G Cap, Kozak sequence NCA-7d (5′ UTR), and signal peptide were placed prior to the peptide sequences. A 120 nucleotides long with S27a+R3U (3' UTR), stop codon and Poly A tail was constructed. The mRNA vaccine construct is graphically illustrated in Figure 6(a).

#### 3.7.2. Multiepitope Subunit Vaccine

The development of a multiepitope vaccine design that is safer for human usage has evolved with the use of computational models in vaccine design. The MSV construct comprised of beta-defensin (adjuvant), 4 CTL, 4HTL, and 6 LBL epitopes joined together using suitable linkers. To boost immune response, beta-defensin was added at the N terminal of the vaccine construct using EAAAK linker. Another sequence, PADRE, is placed between adjuvant and epitopes using the GGGGS linker. Furthermore, AAY, PMGLP, and GGGGS linkers were added to join CTL, HTL, and LBL epitopes together. These linkers stop the self-folding of the epitopes and allow the vaccine construct to stabilize and boost the immune response. The final MSV construct was 317 amino acids in length. The MSV construct is graphically presented in Figure 6(b).

### 3.8. Physiochemical Properties of MSV

To assess the antigenic potential of the MSV, VaxiJen server was used; however, to ensure the nonallergenicity of the vaccine construct, Algpred2.0 server was used. The results ensured the MSV construct is antigenic and nonallergenic. Furthermore, molecular weight, PI, half-life, aliphatic index, and GRAVY were calculated using ProtParam server. The antigenic, nonallergenic, and other physicochemical properties are presented in Table 6.

### 3.9. MSV Secondary Structure Prediction

**Figure 7:**
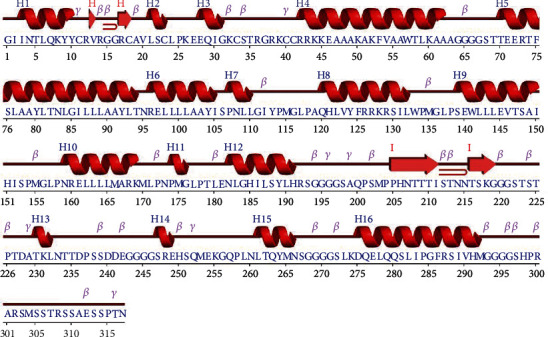
29.97% alpha helix, 16.09% extended strand, and 53.94% random coils predicted by SOPMA server.

SOPMA server predicted that the MSV has 29.97% alpha helix, 16.09% extended strand, and 53.94% random coil. The secondary structure of the MSV is presented in Figure 7.

### 3.10. MSV 3D Structure Modeling, Refinement, and Validation

**Figure 8:**
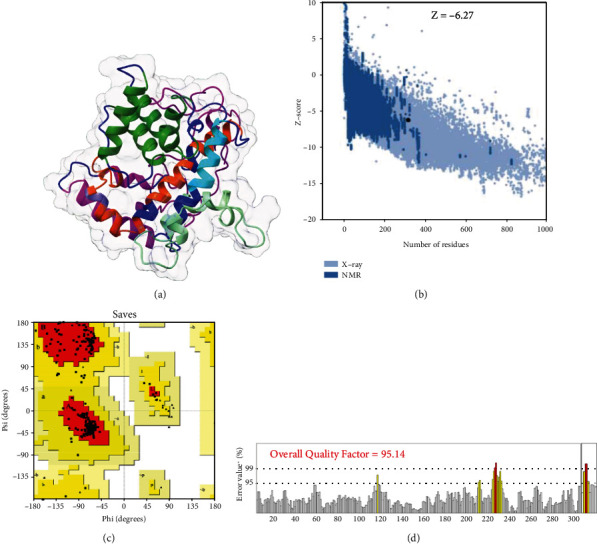
The refined 3D model of the multiepitope subunit vaccine. (a) The 3D model of the MSV where the adjuvant (lime green), PADRE (cyan), CTL epitopes (orange red), HTL epitopes (forest green), and LBL epitopes and linkers (blue) are depicted. (b) The *Z*-score predicted by ProSA-web. (c) The R plot generated by Procheck and (d) quality factor calculated by the ERRAT server.

Model 1 was selected from Robetta for further processing using GalaxyRefine server. The best one based on several parameters, GDT-HA (0.98), RMSD (0.30), MolProbity (1.61), clash score (12.6), poor rotamers (0.4), and R plot (98.1), was validated using several tools (ERRAT, ProSA-web, and Procheck). ERRAT, ProSA-web, and R plot calculated by Procheck were used to estimate the quality of refined MSV model (Figure 8(a)). The ProSA-web calculated a quality score of -6.27 (Figure 8(b)). The Ramachandran plot generated by Procheck server showed that 91.1% of residues lie in the most favorable areas (A, B, and L regions in the R plot), 8.9% in additionally allowed areas (a, b, l, p), and none of the residues were within generously allowed and disallowed regions (Figure 8(c)). The ERRAT score was found within the acceptable range of 95.14 (Figure 8(d)).

### 3.11. Vaccine-TLR Docking

**Figure 9:**
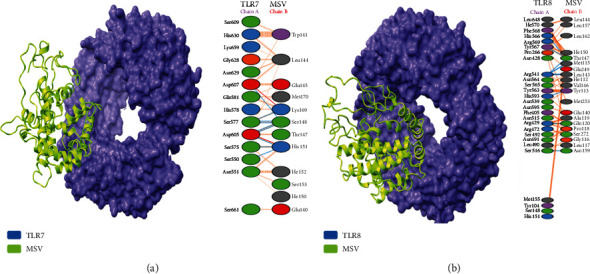
(a) The MSV-TLR7 complex, where the MSV and TLR7 are presented in green and blue colors, respectively. The residual interaction maps of MSV-TLR7 are provided to the right. (b) The MSV-TLR8 complex, where the MSV (green) and TLR8 (blue) are presented. The residual interaction maps of MSV-TL8 are provided to the right.

PyDock server was used for in silico molecular docking of MSV with TLR7 (PDB ID: 7CYN) and TLR8 (PDB ID: 6ZJZ), whereas PDBsum was used to explore interaction analysis of MSV-TLR complexes. The PDBsum interaction analysis revealed the 2 salt bridges and 9 hydrogen bonds, while 227 are nonbonded contacts. The complex formed ten hydrogen bonds between chain A (MSV) and chain B (TLR7) Ser550-His151, Ser575-His151, Ser577-Lys169, Ser577-Ser148, Ser577-Ser148, Asp605-Ser148, Asp605-Ser148, Asp605-His151, and Asp607-Ser148 residues. The two salt bridges were formed between Asp605-His151 and Asp607-Lys169 residues. Furthermore, 10 hydrogen bonds and 243 nonbonded contacts were reported between MSV and TLR8, Arg429-Gln-120, Ser516-Asn-159, Arg541-Met-115, Arg541-Leu-143, Arg541-Thr-147, Arg541-Met-115, Arg541-Leu-143, His566-Thr-147, Tyr567-Thr-147, and His593-Tyr-113 residues. The molecular docking and interchain residual interaction maps of MSV-TLR7 and MSV-TLR8 are provided in Figures 9(a) and 9(b), respectively.

### 3.12. In Silico Immune Response

**Figure 10:**
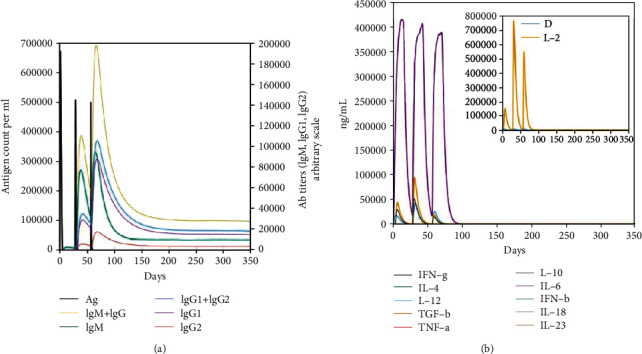
In silico simulation triggered by mRNA vaccine. (a) Antigen count per ml for 3 doses. (b) Ab response to antigen injections.

Increased immune response was observed in 2^nd^ and 3^rd^ doses as compared to the first dose of the vaccine which represent real-world scenario of an ideal vaccine (Figure 10). The immunoglobulin (Ig) M levels were considerably higher than IgG. The secondary and tertiary responses show higher concentration of immunoglobulins, while the concomitant antigen reduced with time. This response is an indicator memory formation against the target pathogen, in this case MARV. Memory formation as observed in immune simulation will prevent future infections by MARV. In addition, the immune simulation results also indicates that some B cell isotypes might also stay for a long time, hinting that isotype flipping and memory formation are possible. In response to each population's individual memory development, the CTL and HTL populations showed a similar growth. Furthermore, dendritic cell activity remained constant while macrophage activity rose. Levels of IFN- and IL-2 were also raised. The innate immune system's elements, including epithelial cells, were also activated. A low Simpson index (D) suggests a wide range of immune activation.

## 4. Discussion

Marburg virus causes severe and often fatal hemorrhagic fever in humans and also in nonhuman primates. Given its high rate of mortality and morbidity, there is a pressing need for the development of vaccine to provide immune protection against it. In recent years, advances in immunoinformatics and reverse vaccinology have enabled researchers to design and develop vaccines against viral diseases using novel approaches. Immunoinformatics-based vaccines offer several advantages over conventional vaccines. It allows the identification of specific antigens that are unique to a particular pathogen. This precision makes it possible to design vaccines that target only the most relevant antigens. This is in contrast to conventional vaccines, which often contain a mixture of antigens, some of which may not be as effective in eliciting an immune response. Another advantage over conventional vaccine is that it enables the rapid identification of potential vaccine candidates. This is particularly important in the case of emerging infectious diseases, where a fast response is necessary to contain an outbreak. In contrast, conventional vaccine development can take years to identify the most effective antigens and optimize the vaccine formulation. Major advantage of the immunoinformatics-based vaccines is that it can be customized for specific populations or individuals. For example, a vaccine can be designed to target specific variants of a pathogen that are prevalent in a particular region. This customizability is not possible with conventional vaccines, which are often designed to target a single variant of a pathogen. This study focuses on the development of an mRNA vaccine against Marburg virus. Among many types of vaccines where each has its own pros and cons, mRNA-based vaccines have gain popularity after success of Moderna and Pfizer vaccines in COVID-19 pandemic. mRNA-based vaccines are more effective since they are comprised of key regions of antigenic proteins of the pathogen. mRNA vaccines made against HIV-1, Zika virus, rabies, influenza virus, and SARS-CoV-2 (Moderna and Pfizer) represent the success of mRNA-based vaccines, and the aforementioned examples of mRNA vaccines represent an efficient subgroup of such vaccines from the first successful mRNA-based vaccination in 1990. Two vaccine candidates, a multiepitope subunit vaccine that must be synthesized outside the host body and mRNA vaccine candidate that must be synthesized inside host cell machinery, were designed in this study. TCRs recognize antigens that are visible on the surface of APCs, or antigen-presenting cells, which are joined to class I and class II MHC molecules. The cytotoxic and helper T cells, correspondingly, identify these antigens. BCRs recognize and respond to antigens on viral proteins that are accessible on their surface, releasing antibodies and commencing humoral host defenses [45, 59].

For instance, researchers are very interested in computational approaches to reveal the molecular basis and identify novel therapeutics, vaccines, or peptides for various infections [15, 53, 60–62]. Bioinformatics and immunomics came together to establish the field of immunoinformatics. This science's major objective is to examine an organism's proteome and anticipate certain immunological responses. The “immunome,” which is made up of all the controlling genes and proteins involved in an organism's immune response, must be understood in order to perform these immunological-based analyses. High-throughput technology has recently advanced immunomics research, enabling the visualization of the immune system's fundamental regulatory processes. This also highlights the possible effects of techniques based on reverse vaccine development for developing vaccines against human infections. Even while immunological investigation is costly and time-consuming, conventional techniques for vaccine creation also have several limitations. Utilizing computer simulations is essential to overcoming these constraints. Bioinformatics methods may be used in this situation to help manage massive immunological datasets quickly, cheaply, and with a high level of accuracy. Through the use of recombinant DNA technology, such techniques may hasten the epitope screening process and aid the practical usefulness of these vaccine formulations [63, 64]. The development of a multiepitope vaccine layout that is safer for human usage has evolved with the use of computational models in vaccine design. Until now, traditional methods for developing vaccines generally depended on using many proteins or an entire disease as vaccine candidates. These, nonetheless, may have had greater antigenic loading and caused allergies. The development of peptide-based vaccination designs entirely overcame these difficulties. Short peptide segments are employed to create multiepitope-based vaccination constructions in this procedure, together with the appropriate adjuvants and linkers, in order to induce highly specific immune responses and precise targeting. Extremely antigenic epitopes may now be selected using sophisticated computational modeling and included in final peptide-based vaccination formulations. Moreover, genomic approaches and codon adaptation methods have been used to predict specific epitope sequences for designing epitope vaccines against *Tropheryma whipplei* [65]. In addition, such computational methods are implemented against the orthohantavirus to design T cell epitopes [66].

In our study, we used a variety of immunoinformatics tools for prediction and evaluation of B and T cell epitopes. T and B cells epitopes are presented to respective cells of the immune system; the epitopes included in the final vaccine were parts of three major proteins of MARV. The residues of the epitopes give empirical information about how immunogenic the vaccine is and also give information about exposure of the epitope on surface of respective cells. The immunogenicity and surface exposure information is calculated from presence of residues having aromatic side chains [67]. The response of immune system to a virus is dependent on CTL-mediated cytotoxicity. Cells that are infected tend to degrade viral proteins that are then presented on its surface to the CTLs [68]. CTLs are responsible for clearing viral particles by release of specialized cytotoxic granules after recognition of parts of viral proteins, generally known as epitopes [69]. Four CTL epitopes are part of the final vaccine we constructed in this study. Alongside, four HTL epitopes were selected for inclusion based on similar criterion. B lymphocytes bind the epitopes and are detected by HTLs that have the matching T cell receptor [70]. Due to this, B lymphocytes undergo differentiation to become plasma cells that produce antibodies [71, 72]. The produced differentiated plasma cells are key to neutralizing viral particles in host body [73]. Along with producing antibodies to neutralize the virus, plasma cells also formulate the formation of long-lived plasma cells and memory B cells. This information is used in case of next infection [74]. The vaccine constructed in this study has six linear B cell epitopes [70]. Population coverage analysis is one of the most important parameters to evaluate the efficacy of a vaccine candidate in terms of the populations to which it can provide immune protection against a given pathogen. Population coverage analysis of T cell epitopes added to the vaccine sequence was tested against all available HLA data from around the world which revealed the required diverse population coverage. Molecular docking was used to evaluate the interaction pattern of the epitopes and receptors. The lower energy represents stronger ligand interaction with key active site residues of the receptor [75]. The binding scores of our epitopes demonstrate robust binding with their respective receptor.

Optimal spacers were inserted into the mRNA sequence to avoid interaction between domains. It also gives stability to vaccine [76]. At the 5 prime-untranslated region, NCA-7d was placed whereas at the 3 prime end, S27a+R3U was added to vaccine sequence. Both the untranslated regions are known to provide considerable stability to the mRNAs. The Kozak sequence was also added right after the 5 prime NCA-7d [76]. In order to further improve the vaccine's transport efficacy, secretory signals were added; these signals have information about relocation of the translated mRNA vaccine via the endoplasmic reticulum compartments. Addition of secretory signals is known to improve vaccine efficacy by efficient relocation of epitopes to the surface of the cells [77, 78]. In addition to the CTL, HTL and Linear B cell epitopes and Adjuvant (Beta defensin) and a PADRE sequence were added to prepare a multi-epitopes subunit vaccine construct. The MSV's antigenicity allergenicity and physiochemical (MW, PI, half-life, AI, and GRAVY) were found within the acceptable range. The 3D structure of a protein provides an ample amount of information about its stability; Robetta server was utilized to predict 3D mode of the MSV. Validation is one of the crucial steps in protein structure modeling; it identifies key errors in protein structure for further refinement when needed. ERRAT, ProSA-web, and Ramachandran plot (by Procheck) was used to identify errors in the MSV structures, and model 1 generated by Robetta was found to pass all of the threshold validation scores. Using the C-ImmSim server, computational immune simulation was performed which revealed the production of a high level of immune response triggering factors [79, 80]. This vaccine profile suggests that immunological memory development will lead to MARV natural immune defense. This study provides a rationale starting point for the development of an efficient vaccine against Marburg virus; however, the findings need further experimental validation to confirm the computational finding of this study.

## 5. Conclusion

The current framework of the creation of mRNA and MSV candidates has shown to be tremendously advantageous, notably in terms of inducing cell-mediated or adaptive immunity. In this work, immunoinformatics and computationally meta-analysis methods were used to identify possible B cell and T cell epitopes generated from antigenic MARV proteins. It was determined whether certain T cell epitopes could bind to the appropriate HLA molecules and the modelled MSV protein with TLR7 and TLR8, using peptide modeling and in silico molecular docking. The final vaccine candidate validated through various methods could instigate the immune system against MARV, and further verification of its effectiveness using in vitro and in vivo tests is necessary to confirm the vaccine's efficacy against MARV. This research may aid in the development of RNA-based vaccinations, as well as prompt early corrective measures and efficient defenses against MRV.

## Figures and Tables

**Table 1:** Selected antigenic proteins of Marburg virus for B and T cell epitope prediction.

S. no	Protein	UniProt ID	Antigenic score	Amino acid count
1	Envelope glycoprotein	P35253	0.54	681
2	VP24	P35256	0.54	253
3	VP30	P35258	0.56	281

**Table 2:** CTL epitopes presented with respective binding affinity and physiochemical characteristics.

Protein	ID	Peptide	Comb	Antigenicity	Immunogenicity	Solubility	Toxicity
Envelope glycoprotein	577	TTEERTFSL	0.87	1.04	0.22	1.814	-1.35
	652	LTNLGILLL	0.91	1.27	0.12	0.219	-1.12

VP30	101	LTNRELLLL	0.78	1.13	0.12	1.395	-1.07

VP24	136	ISPNLLGIY	1.49	1.05	0.07	0.958	-1.36

Antigenicity greater than 0.4, immunogenic less than 1, solubility greater than 1, and toxicity score lower than 0.

**Table 3:** HTL epitopes are presented with corresponding binding affinity and physiochemical properties.

Proteins	Start-end	Peptide	Percentile rank	Antigenicity	IFN epitope	Allergenicity	Solubility	Toxicity
Envelope glycoprotein	425-439	AQHLVYFRRKRSILW	1.3	1.06	+	0.23	1.04	-1.03

VP30	178-192	NRELLLLMARKMLPN	1.8	0.47	+	0.22	1.77	-1.22
	123-137	TLENLGHILSYLHRS	2.1	0.47	+	0.3	1.34	-1.27

VP24	150-164	SEWLLLEVTSAIHIS	0.6	0.93	+	0.27	0.86	-1.68

Antigenicity threshold > 0.4, IFN-gamma-inducing capability, nonallergenic threshold score < 0.3, solubility score > −1, and nontoxicity score of <0.

**Table 4:** Selected linear B cell epitopes with respective binding affinity score and physiochemical properties.

Protein	ID	Peptide	Score	Antigenicity	Allergenicity
Envelope glycoprotein	329	AQPSMPPHNTTTISTNNTSK	0.99	0.55	0.21
	245	TSTPTDATKLNTTDPSSDDE	0.99	0.45	0.27

VP24	230	REHSQMEKGQPLNLTQYMNS	0.94	0.62	0.22
	102	LKDQELQQSLIPGFRSIVHM	0.917	0.56	0.22

VP30	35	HPRARSMSSTRSSAESSPTN	0.99	0.82	0.26
	35	HPRARSMSSTRSSAESSPTN	0.99	0.82	0.26

Antigenic (>0.4) and nonallergenic (<0.3).

**Table 5:** Selected T cell epitopes with their corresponding HLA molecules.

CTL epitopes				
Protein	ID	Peptide	HLA	PDB ID
Envelope glycoprotein	577	TTEERTFSL	HLA-B^∗^08:01	7NUI
	652	LTNLGILLL	HLA-B^∗^58:01	5VWH
VP30	101	LTNRELLLL	HLA-B^∗^58:01	5VWH
VP24	136	ISPNLLGIY	HLA-A^∗^01:01	4NQX

HTL epitopes				
Proteins	Start-end	Peptide	HLA	PDB ID
Envelope glycoprotein	425-439	AQHLVYFRRKRSILW	HLA-DRB1^∗^15:01	1BX2
VP30	178-192	NRELLLLMARKMLPN	HLA-DRB1^∗^15:01	1BX2
	123-137	TLENLGHILSYLHRS	HLA-DRB1^∗^15:01	1BX2
VP24	150-164	SEWLLLEVTSAIHIS	HLA-DRB1^∗^07:01	6BIJ

**Table 6:** The physiochemical properties of multiepitope subunit vaccine predicted by VaxiJen, Algpred2.0, and ProtParam servers.

Property	Score	Result
Allergenicity	0.1	Nonallergen
Antigenicity	0.7	Antigen
Molecular weight	33 kDa	—
Theoretical PI	9.7	Basic
Formula	C1474H2395N435O448S17	—
Half-life (E. coli)	>10 hrs	—
Aliphatic index	79	Thermostable
GRAVY	-0.33	

## Data Availability

The data used to support the findings of this study are included within the article.
